# Characterization of Progenitor Cells during Canine Retinal Development

**DOI:** 10.1155/2012/675805

**Published:** 2012-02-27

**Authors:** Mallely Ávila-García, Gustavo García-Sánchez, Esmeralda Lira-Romero, Norma Moreno-Mendoza

**Affiliations:** ^1^Departamento de Biología Celular y Fisiología, Instituto de Investigaciones Biomédicas, Universidad Nacional Autónoma de México, Ciudad Universitaria, Apartado Postal 70228, Ciudad de México, DF 04510, Mexico; ^2^School of Veterinary Medicine, Louisiana State University, Baton Rouge, LA 70803, USA

## Abstract

We identify the presence of progenitor cells during retinal development in the dog, as this species represents a natural model for studying several breed-specific degenerative retinal disorders. Antibodies to detected progenitor cells (Pax6, C-kit, and nestin) and ganglion cells (BDNF, Brn3a, and Thy1) were used in combination with H3 for the purpose of identifying proliferating cells. Pax6, nestin, C-kit, and H3 were localized mainly in the neuroblastic layer of the retina during the embryonic stage. During the fetal stage, proteins were expressed in the inner neuroblastic layer (INL) as well as in the outer neuroblastic layer; BDNF, Thy1, and Brn3a were also expressed in the INL. During the neonatal stage only C-kit was not expressed. Proliferating cells were present in both undifferentiated and differentiated retina. These results suggest that, during canine retinogenesis, progenitor cells are distributed along the retina and some of these cells remain as progenitor cells of the ganglion cells during the first postnatal days.

## 1. Introduction

Progressive retinal cell death is a common phenomenon observed in human or animal degenerative eye diseases such as progressive retinal atrophy, age-related macular degeneration, retinal detachment, and glaucoma [1]. Different treatment strategies directed towards medically slowing the progression of retinal degenerative diseases with oral supplements have been considered including preservation of affected retinas with specific neurotrophic growth factors and the implantation of retinal pigment epithelial cells (RPEs) in affected eyes. Currently stem cells are being investigated as a potential cellular source for replacing damaged RPE or photoreceptor cells [2]. Both adult bone-marrow-derived stem cells and embryonic stem cells are being used in animal models with the goal of investigating how to induce appropriate cell integration and differentiation.

 Single pigmented ciliary margin cells which are able to differentiate into retinal-specific cell types have been identified in the adult mouse retina [3]. Stem cells derived from the pars plicata and pars plana of the retinal cell margin of human eyes produced all of the different cell types, demonstrating multipotentiality [4]. Based on these data, it was suggested that the sphere-forming cells in the mammalian ciliary epithelium (CE) are retinal stem cells. However, it was revealed that the clonogenic spheres derived from the mouse and human CE originate from differentiated pigmented CE cells rather than the stem cell population harbored in the CE [5]. Fetal cat retinal sheets incubated with BDNF microspheres were transplanted to the subretinal space to investigate whether it is possible for sheets of fetal retinal allograft to integrate into the dystrophic Abyssinian cat retina where progressive rod cone degeneration is taking place [6]. To the authors knowledge there is a lack of information regarding generation, proliferation, and differentiation of progenitor cells during embryonic and fetal stages in other mammals, particularly in the dog where progressive retinal degeneration associated with different ocular diseases is frequently observed.

Multipotent progenitor cells are stem cells which preserve their capacity and potential for self-renewal, giving rise to cells from multiple lineages; however, these are limited in number [1, 7–9]. During the optic cup stage of the developing embryonic eye, a group of multipotent progenitor cells exist which manifest the ability to differentiate into neurons and glial cells. In some species, these cells are able to differentiate into adult stages of development. The retina of many fish and amphibians grows throughout life, roughly matching the overall growth of the animal. The new retinal cells are continually added at the anterior margin of the retina, in a circumferential zone of cells, known as the ciliary marginal zone, or CMZ. In chickens, has been found that new neurons are added to the retina via proliferation and subsequent differentiation of neurons and glia at the retinal margin in a zone highly reminiscent of the CMZ of lower vertebrates [10]. Likewise, other researchers have reported that it may be possible to isolate putative retinal stem cells from the ciliary margin of the adult mouse [11]. In some birds this capacity is apparently only evident during the embryonic stage, and the types of cells produced by progenitors at the retinal margin can be altered by exogenous growth factors although normally the microenvironment imposes limitations on the types of neurons produced [12–14].

Retinal neurogenesis is largely determined intrinsically. Many genes have been identified in order to determine the fate of specific types of retinal neurons [15]. Some of the genes involved in the fate and maintenance of the progenitor cells at the ciliary margin zone include Shh, Notch, and Wnt as well as Chx10, Pax6, Rx1, Six3, and Six6. Their activation either induces the formation or modifies the presence of specific cell types whereas their loss induces important ocular malformations such as anophthalmia, microphthalmia, cyclopia, coloboma, aphakia, among others [16–18].

 A series of genes express themselves early during ocular development, responsible for determining specific eye fields, critical to the development of the retina [19]. Within this group of genes, the transcription factor Pax6 is considered of utmost importance during eye development, as it has been observed that this actively participates in the differentiation of the lens and retina during embryonic development, whereas in the adult eye its expression is maintained in the lens, cornea, ganglion, and amacrine cells [20]. Mutation of this gene induces the formation of ectopic structures in *Xenopus* and *Drosophila* [21, 22]. The expression of Pax6 in retinal progenitor cells has also been observed in mice where its limited expression leads to a reduction in mitotic activity with delayed differentiation of neurons, and when absent the differentiation of progenitor cells is restricted to the generation of amacrine cells only [23–29]. Recently, the expression of Pax6 was detected in a small number of cell progenitors in the inner neuroblastic layer (INBL) of the chicken [30].

Nestin has also been described as a marker of neural progenitor cells [31]. Specifically it is expressed both in neural and glial cells, as well as in their common precursors [32]. In foetal human retina, nestin expression has been shown to occur in Müller cells [33]. Likewise, cells positive to nestin have been found in adult human retina [34, 35].

Retinal ganglion cells are the first mature retinal cells to differentiate from the immature retinal progenitors. Certain genes such as BDNF, Thy1, and Brn3a have been used as markers of differentiated ganglion cells [36]. Particularly, different authors have reported the expression of BDNF and TrkB in the ganglion cell layer and inner nuclear layer of different species of vertebrates [37–39].

Presently, few reports provide evidence indicating the existence of retinal progenitor cells in adult mammals. In this study, we identify the presence of progenitor cells during retinal development in the dog. This species represents a potential, natural, animal model for studies focusing on a number of degenerative retinal disorders.

## 2. Materials and Methods

### 2.1. Animals

 Embryos, fetus, and neonatal dogs were provided by the Canine Control Center at Ecatepec, State of Mexico, and the Department of Surgical Teaching of the Faculty of Veterinary Medicine and Animal Science at the National Autonomous University of Mexico (UNAM). All experiments were approved by and under the supervision of the Norma Oficial Mexicana NOM-062-ZOO-1999. Before enucleation the animals were euthanized with a lethal dose of sodium pentobarbital (50 mg/kg). Eyes were immediately fixed in 4% cold paraformaldehyde in 0.1 M phosphate-buffered saline (PBS) for immunofluorescence analysis.

### 2.2. Histological Analysis

For histological paraffin sections, enucleated eyes were fixed in 4% paraformaldehyde solution (PFA, Sigma, USA) in PBS (phosphate-buffered saline, pH 7.1, Gibco) for 24 h. at 4°C. Later, ocular samples were dehydrated in a gradual alcohol series, embedded in paraffin (Paraplast, McCormick Scientific), and cut into serial sections (15 *μ*m). The sections were mounted on glass slides, dewaxed, rehydrated, and stained with Hematoxylin and eosin. For sections embedded in plastic resin, the collected eyes were fixed in the Karnovsky solution (glutaraldehyde 2.5% in 0.1 M phosphate buffer, pH 7.4) [40] for 12 h. at 4°C. These were then washed with sodium cacodylate buffer and postfixed with 1% osmium tetroxide (OsO_4_) in Zetterqvist's buffer for 1 h., washed again with distilled water, and dehydrated in a gradual alcohol series and acetronile; to be finally embedded in Epon 812 resin. Semithin sections (1 *μ*m) were obtained with an ultra microtome and then dyed with toluidine blue. Fine sections (60 to 90 nm) were contrasted by applying uranyl acetate and lead citrate for transmission electron microscopy.

### 2.3. Immunofluorescence

 Ocular tissue samples were removed, fixed for 20 min. With 4% paraformaldehyde solution in PBS and cryoprotected with 30% sucrose in PBS at 4°C overnight. Later tissues were embedded in OCT medium (Tissue-Tek, Sakura Finetek, Torrance, CA) and frozen in hexane (J. T. Baker) on dry ice. Serial frozen transversal sections of approximately 15 *μ*m were obtained to be used for immunofluorescence using conventional techniques. Sections were then incubated with the primary antibodies and treated with the secondary corresponding antibody. As a positive control, one day postnatal and adult mouse CD1 eyes were employed. As a negative control, samples without primary antibody were incubated. Subsequently, these were washed with PBS and double consecutive immunofluorescences were carried out, in order to simultaneously detect different proteins. Finally these were mounted in Dako paramount aqueous mounting medium (Dako North America, INC. USA). The antibodies and dilutions used are presented in Table 1. Sections were analysed in a confocal microscope (LSM 5 Pascal, Zeiss) equipped with Argon-Kripton and Helium-Neon laser using filters BP 450–490 and 546/12. Double immunofluorescences were detected simultaneously. 

## 3. Results

The presence and distribution of retinal progenitor cells during embryonic stages (up to 25 days of gestation: dg), fetal (30–60 dg), and neonatal (1 day postnatal: dp) was studied for canine development. These stages were determined according to criteria established by Aguirre et al. [41], Simoens and Budras [42], and Cook [43, 44], and corroborated by histological examination of the retina. 

### 3.1. Morphogenesis of Canine Retina

#### 3.1.1. Embryonic Stage

**Figure 1:**
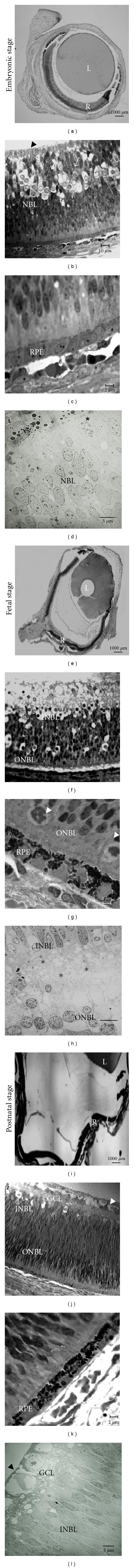
Histological analysis of the retina in embryos ((a)–(d)), fetuses ((e)–(h)) and neonatal ((i)–(l)) dogs. (a) Transversal section of the eye showing the retina (R) and lens (L). (b) In longitudinal section, retina consists of a neuroblastic layer (NBL) formed by an inner marginal (arrow head) and outer nuclear zone (arrows). (c) The retinal pigment epithelium (RPE) is observed to consist of a single layer with oval shaped melanosomes on the apical surface. (d) Electron micrograph showing cells with mitotic activity (*) localized in the NBL. (e) Eye from the fetal stage where retina (R) and lens (L) are evident. (f) Cell migration has formed the inner neuroblastic layer (INBL) and outer neuroblastic layer (ONBL). (g) Some rudimentary photoreceptors were evident at the fetal stage (arrow head). (h) The INBL and ONBL are separated by the transitional space of Chievitz where high resolution exposes a space between the INBL and ONBL without any cells (*). (i) Eye from the postnatal stage where part of the lens (L) and the retina (R) are visible. (j) During the postnatal stage the ganglion cell layer (GCL) was apparent. (k) The RPE was observed as a row of cubic cells. (l) Electron micrograph showing the GCL where the axons (arrow head) are forming the nerve fiber.

 At 25 days of embryonic development, taking histological sections of the eye, there appear to be primary fibers in the lens which are completely separate from the surface ectoderm (Figure 1(a)). At the back of the eye, the retina was found to consist of a neuroblastic layer (NBL) which in turn divided into two parts: an inner marginal area (not nuclear) and another outer nuclear zone (Figure 1(b)). The retinal pigmented epithelium (RPE) consists of several rows of cells with pigment (Figure 1(c)). At electron microscope level, mitotic figures are evident in the inner neuroblastic layer (INBL, Figure 1(d)). 

#### 3.1.2. Fetal Stage

Fetuses between 35 and 55 days of gestation manifested clear development of various structures which make up the retina (Figure 1(e)). Histologically, it was observed that cells from the nuclear area migrated to the internal marginal zone, forming two nuclear layers: the inner neuroblastic layer (INBL), and the outer neuroblastic layer (ONBL, Figure 1(f)). Both layers were separated by the transient layer of Chievitz, evidently a space with no cell content (Figure 1(h)). In the innermost part of the INBL some ganglion cells project their axons in order to initiate the formation of the optic nerve, forming the nerve fiber layer (NFL). The retinal pigmented epithelium (RPE) manifests several layers of cells and in some areas the presence of rudimentary photoreceptor precursor cells (Figure 1(g)). 

#### 3.1.3. Postnatal Stage

In the last stage analyzed, corresponding to 1 day postnatal puppies, the development of the layers is more evident (Figure 1(i)). The retina is formed by the NFL, and a greater number of ganglion cells and axons can be observed directed towards the brain in order to form the optic nerve. The ganglion cell layer (GCL) was shown to be separated from the INBL by the inner plexiform layer (IPL, Figures 1(j) and 1(i)). The RPE manifested a row of cubic cells, and in some of these the microvillus was evident (Figure 1(k)). 

### 3.2. Identification of Retinal Progenitor Cells

#### 3.2.1. Embryonic Stages

**Figure 2:**
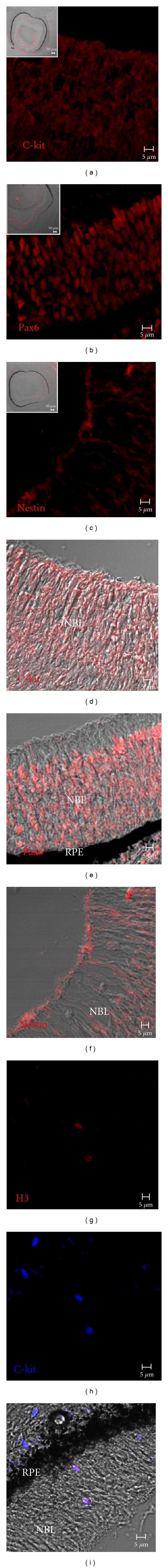
Immunofluorescent staining of C-kit (a and d), Pax6 ((b) and (e)) and Nestin ((c) and (f)) in the retina from dog embryos. The expression was observed in the NBL, examined in combination with Nomarski optics. Inserts present a panoramic view of the pattern of expression for the C-kit, Pax6 and Nestin. (g) Confocal image showing the proliferation of cells stained with H3. (h) The same figure presented in (g) but here the C-kit positive cells are evident. (i) Merged image showing cells expressing both C-kit (blue) and H3 (red) proteins in combination with Nomarski optics.

 At this stage, the expression of C-kit, Nestin, and Pax6 markers were detected located in the lens and the NBL. The expression of C-kit (Figures 2(a) and 2(d)) and Pax6 (Figures 2(b) and 2(e)) was very marked in the cell nuclei. For its part, Nestin (Figures 2(c) and 2(f)) was observed in the filaments of the progenitor cells. The proliferative activity of cells takes place due to the presence of the histone 3 (H3) protein. Thus, we identified an increasing level of expression of H3 in the proliferative zone, both in the central region as well as at the side of the retina. There were also some proliferating cells, located in the marginal layer of the NBL co-localized with cells positive to the C-kit marker (Figures 2(g), 2(h), and 2(i)).

#### 3.2.2. Fetal Stage

**Figure 3:**
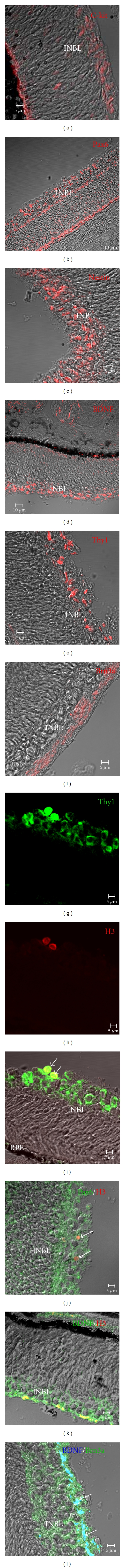
C-kit (a), Pax6 (b) and Nestin (c) expression in the retina from fetal stage. The expression pattern was evident in the INBL. At this stage the differentiation of ganglion cells was evident as were the markers BDNF (d), Thy1 (e) and Brn3a (f). (g) Confocal image showing the Thy1 positive cells. (h) The same image was presented in (g) but made evident with H3. (i) Merged image, but the proliferation of Thy1 cells is evident, localized in INBL (arrows). (j) Merged image illustrates the colocalization of Pax6 positive cells with H3 positive cells (arrows). (k) Merged image illustrating proliferation of BDNF positive cells in the INBL made evident by H3 (yellow stained). (l) Colocalization of BDNF and Brn3a proteins distributed along the INBL (arrows).

During this stage, cells positive to the C-kit (Figure 3(a)), Pax6 (Figure 3(b)), and Nestin (Figure 3(c)) proteins were detected. The marker is primarily located in the INBL, but positive cells were also observed in the ONBL, although they were less intense. Proliferating cells were detected in the proliferative zone located in the ONBL (data not shown). For the identification of progenitor cells with proliferative activity, double immunofluorescence tests were performed using C-kit and Pax6 in combination with H3. Proliferating cells were found in both the INBL as well as in the proliferative zone ONBL. Cells positive both to C-kit and H3 were located in the INBL (Figures 2(g) and 2(h)). Beside this, cell positive to Pax6 demonstrating proliferative activity were located in the INBL (Figure 3(j)).

 Ganglion cell differentiation was evident at this stage of retinogenesis, with the identification of cells positive to BDNF (Figure 3(d)) and Thy1 (Figure 3(e)). The presence of this signal is localized in the cytoplasm of ganglion cells located in the INBL. Contrastingly, only a slight expression of Brn3a in some ganglion cells (Figure 3(f)) was identified. Implementing double immunofluorescence tests using antibodies Thy1/H3 (Figures 3(g), 3(h), and 3(i)) and BDNF/H3 (Figure 3(k)), we observed that certain cells located in the INBL co-localized, and it may prove that these are ganglion cells. The combination of Brn3a/BDNF perfectly delineated the presence of ganglion cells located in the INBL; Brn3a was expressed in the nucleus, whereas BDNF was located in the cytoplasm (Figure 3(l)).

#### 3.2.3. Postnatal Stage

**Figure 4:**
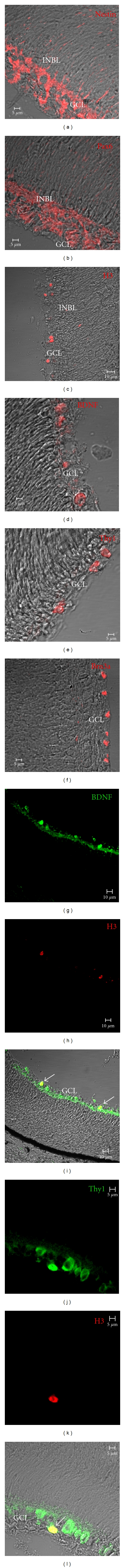
Immunofluorescent staining of Nestin (a) and Pax6 (b) during neonatal stage was observed in the INBL. (c) H3 only revealed in the GCL and did not co-localize with stem cell markers. BDNF (d), Thy1 (e) and Brn3a (f) staining showed the typical morphology of ganglion cells localized in the GCL (red cells). A number of cells marked with BDNF ((g)–(i)) and Thy1 ((j)–(l) were evidently proliferating as they were positive to H3 protein (arrows).

At this stage of retinal development, the expression of C-kit was not observed however the expression of Nestin (Figure 4(a)) and Pax6 (Figure 4(b)) was evident mainly in the ganglion cell layer, whereas limited expression was detected in the both INBL and ONBL. Some cells located in the GCL presented proliferative activity made evident by the positive expression of H3 (Figure 4(c)). BDNF, Thy1 and Brn3a were evident in the GCL, both in the central and peripheral area of the retina (Figures 4(d), 4(c), and 4(f)). The presence of proliferating ganglion cells is also indicated by the colocalization of BDNF/H3 protein (Figures 4(g), 4(h) and 4(i)) and Thy1/H3 (Figures 4(j), 4(k), and 4(l)).

## 4. Discussion

### 4.1. Histological Analysis

The light and electron microscopy in the embryonic stage showed that the retina had a neuroblast layer, perfectly delimited into two zones: inner marginal and outer nuclear zone. The cells exhibited a distinct pattern: prophase and metaphase profiles were similar to those previously described by J. W. Hinds and P. L. Hinds [45]; these findings suggest interkinetic nuclear migration, where cells migrate from the apical side to the basement of the neuroretina, as the cell cycle progresses [46, 47]. The retinal pigment epithelium appeared as a single layer with melanosomes of oval-shape near the apical surface. Taking into account these findings and comparing them with those reported by Aguirre et al. [41] and Cook [43, 44], it was determined that embryos included in this stage had completed 25 days of gestation.

 During the fetal stage, as a result of cell migration in the retina, there were two neuroblastic layers, inner and outer, separated from each other by a cell-free space called the transitional space of Chievitz. At this stage the initiation of cellular differentiation was evident; some cells, in the inner neuroblastic layer (INBL) had differentiated into ganglion cells and their axons established the layer of nerve fibers and the optic nerve. Beside this, some rudimentary photoreceptors became apparent in the outer neuroblastic layer, adjacent to the pigmented epithelium. These findings are consistent with the histological work done by Aguirre et al. [41] and Cook [43, 44] for the fetal embryonic stage in this species. Using electron microscopy, it has been noticed a difference between the nuclei of the inner and outer neuroblastic layers [48]. In the current study these differences were also apparent: in the outer neuroblastic layer the nuclei were oval, in contrast to those of the inner neuroblastic layer where the nuclei were spherical.

At the neonatal stage, histologically the canine retina presents the same characteristics as those described for the fetal stage; however, there are a greater number of ganglion cells. The structure of the ganglion cells was observed using electron microscopy: these cells have very large nuclei which cover the greater part of the cell body and their axons form the nerve fiber layer. At this stage, the retinal pigment epithelium consists of a row of cuboidal cells. These findings were similar to those described by Spira and Hollenberg [48] and Cook [43].

### 4.2. Characterization of Stem Cells

Different markers of progenitor cells have been used with great success for the purpose of identifying these cell types. Pax6 is a key gene during development of the eye, and is actively involved in the differentiation of the lens and retina in different species of vertebrates. Mutations in this gene lead to the presence of different disorders during development, resulting in anomalies of the iris, retina, lens, and cornea. During adulthood, expression continues in lens and cornea, as well as in amacrine cells and retinal ganglion cells [24, 27]. During these three stages, we observed that Pax6 presented a similar pattern covering both the central retina and the peripheral area however its expression was higher in the INBL during the fetal and neonatal stages, similar to findings reported by Doh et al. [30] who noted the expression of Pax6 in chicken embryos; this gene was detected in progenitor cells in the INBL during E4, and, as development advanced, its increase was evident in the inner nuclear layer (CNI). Finally, during late stages of development, both ganglion and amacrine cells expressed this gene.

Nestin is a protein made up of intermediate filaments which can be employed as a marker of neural progenitor cells. It plays an important role in cell movement, including displacement, contraction and cytokinesis [49]. During chicken embryonic development, transitin, which is a homology of Nestin, may be induced in the Müller cells by acute retinal damage. Transitin-positive cells were observed mainly in the ganglion cell layer (GCL) and nerve fiber layer (NFL), possibly marking the endings of the Müller glia; however, intense immunoreactivity was presented in the ciliary margin zone (CMZ) and the ciliary body. Downregulation was restricted to the CMZ prior to hatching. The expression of Nestin in the retina of the dog had a pattern similar to that reported by Ghai et al. [50] in the chicken, where the immunoreactivity was higher in the GCL and NFL. There was, however, a higher expression near the ciliary body, ruling out the existence of a niche as occurs in the chicken. Thus it appears that this niche does not appear in the dog and that the expression of stem cell markers decreases during development.

Another marker of retinal progenitor cells is C-kit, whose participation is widely studied in gametogenesis, hematopoiesis, melanogenesis, and neurogenesis, where this gene has been observed to be involved in the establishment of neural connections. Likewise, C-kit promotes survival, proliferation, and cell differentiation [51, 52]. C-kit has been localized in the neural retinal, pigmented epithelium and ciliary body; yet the expression pattern during retinogenesis is not well known. It has been reported the presence of C-kit in the NBL and GCL during the embryonic stage of the mouse [53]. During the postnatal stage they observed a dramatic decrease, which is consistent with exit from the cell cycle and the initiation of differentiation. In this study we looked at the expression of C-kit protein during embryonic and fetal stages, where positive cells were localized mainly in the inner neuroblast layer (IN); however, it was not found during the neonatal stage. These results are consistent with those described by Koso et al. [53].

An important feature associated with the presence of stem cells is mitotic activity: stem cells in the adult are capable of mitotic activity but are frequently quiescent, whereas progenitors are often very mitotically active. In the case of the retina, it has proposed a model of nuclear migration during the development of the retina, where the nuclei of neuroepithelial cells migrate from apical to basal and basal to apical as the cell cycle progresses [47]. This places cells in G1, G2, and M in the apical zone, adjacent to the pigmented epithelium, whereas cells in phase S remain in the basement zone. It has observed that the expression of H3 starts during the G2 phase and peaks during the metaphase of mitosis [54]. In the present study, we found the presence of proliferating cells located near the retinal pigmented epithelium, confirming that cell migration occurs as part of progression in the cell cycle. In the embryonic stage, proliferating progenitor cells were observed in the germinal zone located near the pigmented epithelium.

### 4.3. Ganglion Cell Differentiation

During cell differentiation, retinal progenitor cells begin to express genes such as Brn3a, Thy1, and BDNF, the exact role of which is imperfectly understood, however, they may all play an important role during the differentiation of progenitor cells to become retinal ganglion cells. In this study, we discovered the expression of three markers in GCC during the fetal stage, by applying immunofluorescence. Although it has been reported that ganglion cells are abundant in the central zone, we observed the presence of ganglion cells in the peripheral and central zones. 

Schlamp et al. [55] commented that in the case of marker ganglion cells in both the mouse and rat with damage to the retina, when expression diminished, damage increased. On the other hand, it has been detected Thy1 and Brn3a in the adult mouse which are identified in the CGL of the ganglion cell body as well as in their dendrites on the inner plexiform layer [56]. Recently, Germanà et al. [39] revealed the presence of BDNF in zebrafish by applying peroxidase immunohistochemistry. They detected the expression of this antibody between 10 dpf and 180 dpf in the outer nuclear, outer plexiform, and inner plexiform layer, observing an increase of similar intensity advancing with development. The expression of Brn3a was observed too in the retina of the chicken which progressed with development, observing that this label was clearly evident in the inner nuclear layer, ganglion cell layer and nerve fiber layer [30].

The expression of ganglion cells was only detected in the fetal and neonatal stage, a result similar to that described by Schlamp et al. [55] and Raymond et al. [56]. Germanà et al. [39] and Doh et al. [30] marked the ganglion cells with the same antibodies used in this work, and likewise we observed the colocalization of ganglion cells with H3. These data suggest that ganglion cells undergo proliferating activity during development.

## 5. Conclusions

Canine retinal progenitor cells express the markers Pax6 and nestin in the neuroblastic layer during pre- and postnatal stages. However, the expression of C-kit was limited to the embryonic and fetal stage probably due to cellular differentiation. Cells manifesting mitotic activity were detected in the embryonic and fetal stages in combination with cells expressing C-kit and Pax6, in contrast to the newborn stage, where the mitotic activity was observed in ganglion cells marked with BDNF and Thy1. The presence of the Brn3a, Thy1 and BDNF proteins were all detected during the fetal stage corresponding to the beginning of cell differentiation. Therefore, we suggest that a small group of progenitor cells localized in the GCL are in fact ganglion cells which are present in the retina of the dog during the first days after birth.

## Figures and Tables

**Table 1:** Antibodies and dilutions.

Antibody	Brand	Dilution
Rabbit polyclonal to PAX6	Abcam, MA, USA (ab5790)	1 : 250
Rabbit polyclonal to Nestin	Abcam, MA, USA (ab5968)	1 : 250
Rabbit polyclonal to C-kit	Santa Cruz Biotechnology, CA, USA (sc-168)	1 : 200
Rabbit polyclonal to BDNF	Santa Cruz Biotechnology, CA, USA (sc-546)	1 : 250
Rabbit polyclonal to BRN3A	Abcam, MA, USA (ab23579)	1 : 50
Mouse monoclonal to Thy1.1	Abcam, MA, USA (ab65193)	1 : 200
Rabbit polyclonal to Histone 3 (H3)	Upstate, Temecula CA, USA (06–570)	1 : 200
Anti-rabbit IgG TRITC conjugate	Zymed, San Francisco, CA, USA (81–6114)	1 : 100
Anti-rabbit IgG Cy 5 conjugate	Zymed, San Francisco, CA, USA (81–6116)	1 : 100
Anti-rabbit IgG FITC conjugate	Zymed, San Francisco, CA, USA (81–611)	1 : 100
Anti-mouse IgG TRITC conjugate	Zymed, San Francisco, CA, USA (81–6714)	1 : 100
Anti-mouse IgG FITC conjugate	Zymed, San Francisco, CA, USA (61–6511)	1 : 100

Primary and secondary antibodies used for immunofluorescence. All dilutions were prepared in *bovine serum albumin.*
